# ROS-induced dramatic lipid changes in *Arabidopsis*

**DOI:** 10.1080/13510002.2021.2002001

**Published:** 2021-11-10

**Authors:** Tianlin Jin, Xue Wang, Zhuying Deng, Xiaofang Liu, Dacheng Liang

**Affiliations:** a Engineering Research Center of Ecology and Agricultural Use of Wetland, Ministry of Education/Hubei Key Laboratory of Waterlogging Disaster and Wetland Agriculture, Jingzhou, People’s Republic of China; b Hubei Collaborative Innovation Center for Grain Industry, Jingzhou, People’s Republic of China; c School of Agriculture, Yangtze University, Jingzhou, People’s Republic of China

**Keywords:** Membrane, lipid, *Arabidopsis*, H_2_O_2_, ROS, ROS inhibitor

## Abstract

**Objectives:** The beneficial role of ROS was probably in promoting intercellular communication by modifying membrane constituents [Liang D. A salutary role of reactive oxygen species in intercellular tunnel-mediated communication. Front Cell Dev Biol. 2018;6:2]. We investigated how the membrane lipids were responding to ROS and ROS inhibitors.

**Methods:** To examine how ROS affected the lipid profiles, we used thin-layer chromatography to characterize lipid profiles in *Arabidopsis* plants. Then, the confocal microscopy imaging was used to confirm the change of membrane lipid in a plasma membrane marker line exposed to ROS and ROS inhibitors.

**Results:** We found the relative contents of most lipids in H_2_O_2_-treated *Arabidopsis* plants were increased in roots, rather than in shoots. The increased fluorescent signal of membrane marker induced by H_2_O_2_ was mainly enriched in the conductive parts of roots. Several ROS inhibitors also strongly affected the lipid profiles. Among them, diethyldithiocarbamate (DDC) can progressively change the lipid profiles with treatment going on. Membrane marker signal was mainly accumulated in the root tips and epidermal cells after treatment by DDC.

**Discussion:** H_2_O_2_ may enhance intercellular communication by inducing different lipid species in the conductive parts of roots. The lipid profiles were widely responding to various ROS reagents and might play a role in intercellular signaling.

## Introduction

Lipids are one of the four major bio-macromolecules that serve as energy storage, basic components of cell membranes as well as signaling molecules. Many important biochemical processes were based on lipid-supported layers or the membranes; for example, photosynthesis, the essential energy source for most life occurs on the thylakoid membrane made of lipids, and aerobic respiration occurs on specialized membrane made of lipids in mitochondria. There are many different types of lipids existing in membranes, the most abundant of which are glycerides present in all membranes, followed by sterols and sphingolipids in plasma membrane [1].

The diverse and abundant lipids composing cell membranes are constantly exposed to environmental stressors. For example, the reactive oxygen species (ROS) generated by many environmental factors including heavy metals, solar radiation, damages etc. present as one of the key challenges to membrane integrity. Under oxidative stress, ROS-induced lipid peroxidation leads to many adverse impacts on membrane structures including increased water leak, decreased lipid bilayer thickness, loss of lipid asymmetry and loss of membrane order [2].

Nevertheless, ROS can also be used as signaling molecules. One of the main benefits of ROS production in plants and animals are facilitating intercellular channel formation [3] for which the importance of ROS-induced membrane protrusions may loom large. From this conclusion, we may hypothesize that the interference of ROS balance might result in the changes of lipid profiling. To test this, we used thin-layer chromatography (TLC) and membrane marker line to monitor cellular lipid changes and found that lipid constitution and/or profiling were indeed significantly changed by hydrogen peroxide and ROS inhibitors.

## Materials and methods

### Plant growth and treatment

Wild-type *Arabidopsis* plants (Col-0) were germinated on MS culture medium. The growth condition was set at 24°C, 15 h/9 h light–dark cycle with light intensity of 90–120 µE m^−2^ s^−1^. For H_2_O_2_ treatment, 7-day old plants were transferred to medium containing 2 mM H_2_O_2_ and treated for 7 days. For the evaluation of H_2_O_2_ concentration, 7-day old *Arabidopsis* seedlings were transferred onto new plates supplemented with H_2_O_2_ at 0, 0.2, 2 or 3 mM, and then left for 4 days at the growth room. Root and leaf tissues were harvested respectively and stored in −80°C freezer for further analysis. For the evaluation of NAA or BFA-induced plasma membrane endocytosis or exocytosis, 7-day old *Arabidopsis* seedlings (*AtPIP2a-mCherry* transgenic lines) were pretreated with 2 mM H_2_O_2_ for 1 day. The pretreatments were followed by 30 min of concomitant treatment with 10 μM BFA, 5 μM NAA or both. BFA was dissolved in dimethylsulfoxide (DMSO) at 10 mM and was diluted to a final concentration of 10 μM with MS medium. Control treatments were used an equal amount of DMSO.

### Lipid extraction

For lipid extraction, samples in the freezer were allowed to warm to room temperature. The samples were weighted (at least 10 mg) and homogenized in 100 µl 0.05 M Tris-HCl (pH 7.4) in a 1.5 ml centrifuge tube with a microcentrifuge motor and pestle. 300 µl chloroform/methanol (2/1, v/v) was added into homogenate. The homogenate-containing tube was vortexed (about 15 s) and settled at room temperature for 10 min. After it was briefly vortexed again, it was centrifuged at 8000 rpm for 10 min to separate the aqueous phase. The lower phase containing the lipid fractions was collected. The chloroform/methanol (2/1) extraction was repeated two additional times and the lipid-containing phases were pooled together. The samples were dried by vacuum freeze drier (Christ ALPHA 1–2 LD plus). Once completely dried, the samples were added chloroform/methanol (1/1, v/v) containing 0.1% BHT (2,6-Di-tert-butyl-4-methylphenol) to re-dissolve the sample according to the standard of 0.1 mg/µl and store at −20°C for further analysis.

### Thin-layer chromatography

To start the TLC experiment, a 100 × 200 × 0.25 mm silica gel coated TLC plate (GF254) was baked in an oven at 50°C for 30 min. The TLC chamber was prepared by adding all the components of the solvent mix (chloroform/methanol/acetic acid [90/10/1, v/v]), then covered and mixed vigorously. Sticking a filter paper on the inner wall of the chamber could facilitate the balance of solution vapor in the chamber. The chamber was left to equilibrate for 20 min before putting the spotted plate in the chamber.

After the baked plate was cooled down to room temperature, a glass minicap was used to spot the samples on a straight line (2 cm from bottom edge of the plate). The total lipids (10 µl) from 1 mg samples were spotted within a diameter of 5 mm. 100 ng standard sample was loaded as a reference. The spotted plate was allowed to air dry for about 5 min. The plate was run in the first mobile phase (chloroform/methanol/acetic acid) for about 2 min so that the solvent front reached 2.5 cm high above the start line. The plate was taken out to allow it air dry. The second mobile phase (hexane/diethyl ether/acetone [60/40/5, v/v]) was prepared as the first one. The dried plate was continuously run till the solvent front reached 9 cm above the start line (approximately 20 min). The plate was air-dried and then run in a third mobile phase (hexane/diethyl ether [97/3, v/v]) to 10 cm above the start line (approximately 25 min).

For lipid staining, two different staining methods were compared. In the traditional method, the developed TLC plate was washed with distilled water for 2 × 10 min, then immersed in oxidizing solution [0.02% (w/v) periodic acid/0.09% (v/v) acetic acid] for 30 min. The oxidized plate was washed with 0.1% (w/v) sodium metabisulfite in 1 × M HCl for 2 × 5 min, and then incubated in Schiff’s Fuchsin-sulfite reagent for 15 min. The plate was washed with 0.1% (w/v) sodium metabisulfite solution in 1 M HCl for 5 min, and the colored spots can be recorded for analysis.

In the second method, the TLC plate was sprayed with a solution of 8% cupric sulfate in 10% aqueous phosphoric acid and air dried for 15 min. The plate was heated in an oven and the programmed temperature was as follows: start 75°C, ramp 5°C/min to 125°C. After the spots were visible, the plate was ready for an image capture (Nikon D7200).

### Membrane marker line generation

To generate plasma membrane marker line, the plasmid (Catalog # 61180, addgene) containing fluorescent reporter for plasma membrane AtPIP2a-mCherry [4] was transformed into *Agrobacterium tumefaciens* strain GV3101. Total 12 independent T1 transgenic *Arabidopsis* lines were produced. Single copy T-DNA insertion line was determined by QPCR and used for further analysis. Stable and consistent fluorescent marker lines were selected in T3 generation and used for our study.

### Confocal microscope

Confocal images were taken with a Leica TCS SP8 confocal microscope (Leica Microsystems, Germany) with a 40 × water-immersion objective and a pinhole of 1 airy unit. The power of laser was set at 9% of its maximum power. For fluorescence excitation, an optically pumped semiconductor laser (OPSL) with output at 552 nm was used. The power supply of the PMT detector was set to 666 V. The PMT detector was set to a range of 570–610 nm. Z-projections of confocal stacks and quantifications of pixel intensities were analyzed by the Leica Application Suite X (LAS X).

## Results

### Leaf and root tissues have different lipid profiles

**Figure 1.**
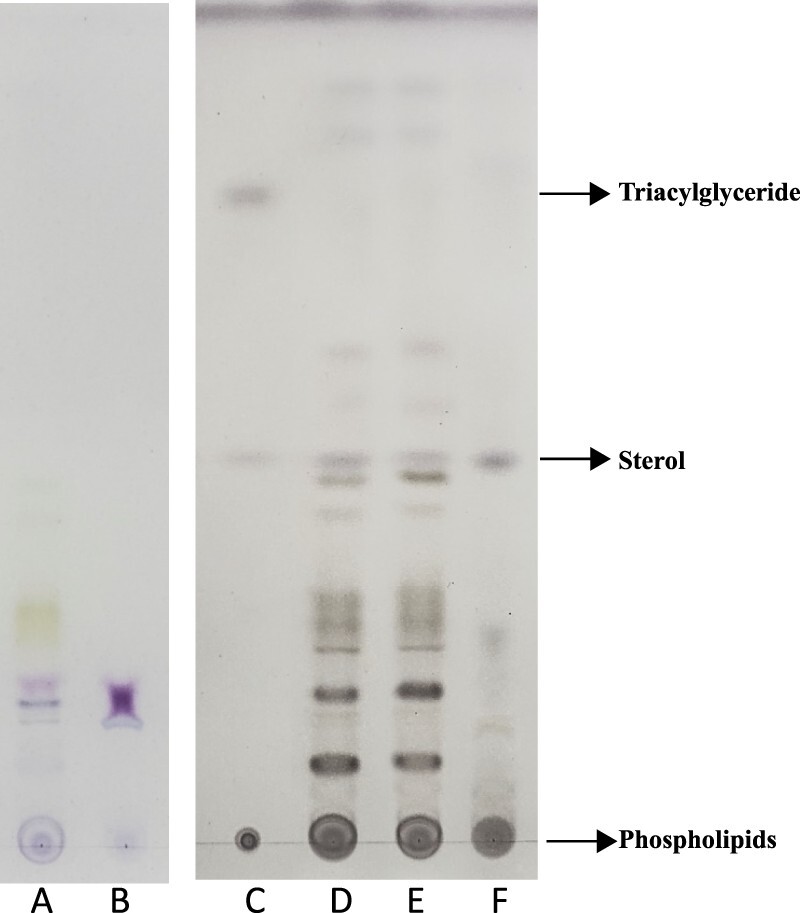
Lipid profiles in leaf and root tissues. Left plate (lane A, B) was stained by Schiff’s Fuchsin-sulfite reagent, right plate (lane C, D, E, F) was stained by cupric sulfate/phosphoric acid. Lane C was standard sample. Lane A and E were lipids from leaf samples. Lane D was from the whole plant samples. Lane B and F were lipids from root tissues.

Plants consist of two distinct tissues: the photosynthetic shoots that convert solar energy into chemical energy, and the non-photosynthetic roots that are specialized in water and nutrient uptake. Since many studies have shown that photosynthesis is tightly associated with lipid biosynthesis [5–7], we wondered if the synthetic and non-synthetic organs had different lipid profiles. To do this, we used two different staining methods to characterize the lipid profiles of leaves and roots: the Schiff’s Fuchsin method and culpric-staining method. As shown in Figure 1, the species of lipids in the leaves were more diverse and also abundant than in the roots using two different staining methods, suggesting leaves and roots were indeed composed of different lipids.

### Hydrogen peroxide treatment and its impact on lipid profiling

**Figure 2.**
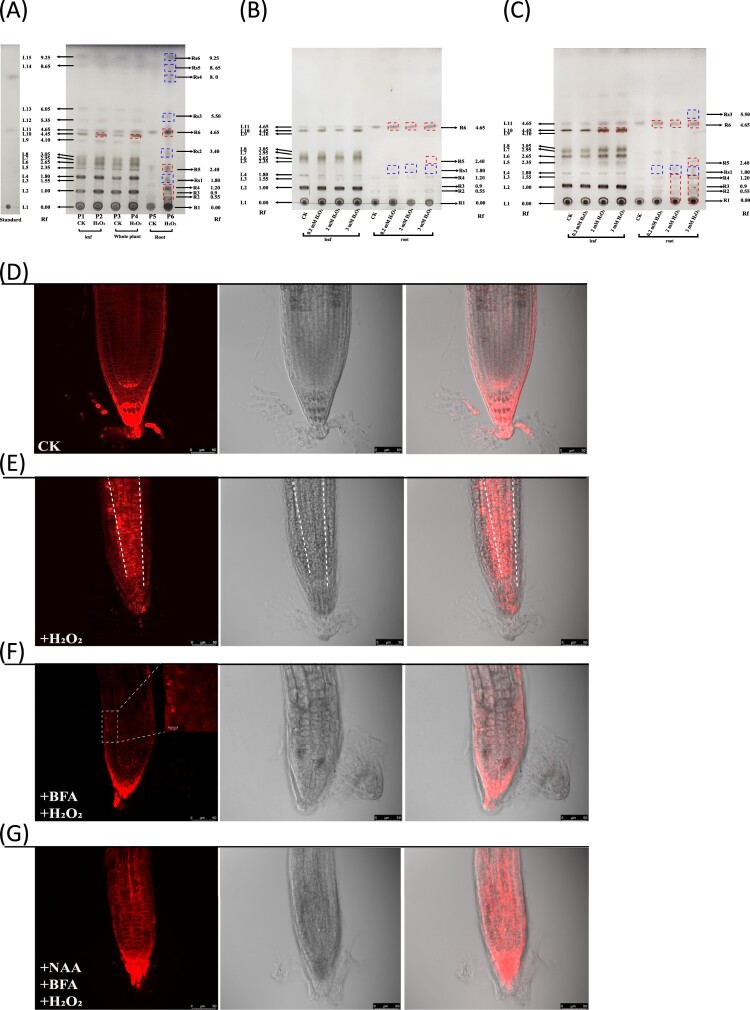
H_2_O_2_-induced lipid profile and plasma membrane fluorescence alteration in root tissues. (A) TLC chromatogram showed all lipid spots observed in whole plant, leaf and root samples after 7-day treatment. Lane P1 and P2: leaf samples; Lane P3 and P4: the whole plant samples; Lane P5 and P6: root samples. Lane P1, P3 and P5 were used as a control; Lane P2, P4 and P6: H_2_O_2_-treated samples. L1: phospholipids; L11 or R6: sterol; Rs4: triacylglyceride. Spots L6-L8 could only be detected in leaf tissues. Rf: retention factor. (B) TLC chromatogram showed lipid profiles of leaf and root tissues under different H_2_O_2_ concentrations after 2-day treatment. (C) TLC chromatogram showed lipid profiles of leaf and root tissues under different H_2_O_2_ concentrations after 4-day treatment. The dashed red box indicated the increased lipid after H_2_O_2_ treatment. The dashed blue box indicated the new spots induced by H_2_O_2_ treatment. The image of (A), (B) and (C) was the representative of three independent TLC running experiments. (D) Confocal imaging of plasma membrane marker line in the control plants. Noted that the fluorescence was mainly distributed in the membrane. (E) Membrane fluorescence was increased after one day of H_2_O_2_ treatment. Noted that the central part of root tissues showed increased membrane marker signal. (F) The H_2_O_2_-induced membrane fluorescence increase can be inhibited by vesicular exocytosis inhibitor BFA (10 μM). (G) Auxin NAA (5 μM) alleviated the BFA-induced internal accumulation of plasma membrane fluorescence. All the plants were imaged in the Leica TCS SP8 confocal platform at the same setting. The 552 nm excitation laser was set at 9% of its maximum power, and the gain of photomultiplier tubes (570 nm –610 nm) was set at 666 V for collecting signal in all the recordings. This figure was representative of 12 different samples in three experiments.

Since the cupric sulfate/phosphoric acid staining was more sensitive in revealing the main lipids such as triglyceride, sterol and phospholipid (Figure 1), we used this method to test whether the spot pattern could be changed when plants were subjected to stress. H_2_O_2_ was used to treat plants given it plays various roles in stress signaling and intercellular communication [3]. As shown in Figure 2(A), more than 20 lipid spots (L1-L15, R1-R6) were revealed after a 7-day course of H_2_O_2_ treatment. Three of the 20 spots were identified in accordance with R_f_ of known standard samples as phospholipids (L1), sterol (L11 or R6) and triacylglyceride (Rs4) respectively. In these spots, L6-L8 spots only appeared in leaf tissues and also appeared green or blue–green before staining. Presumably, they could be derived from photosynthesis-related process.

The total lipid content in the H_2_O_2_-treated samples was not significantly different from that in the control. Only L10 spot was slightly higher in the treated leaf samples (Figure 2(A)), otherwise the lipid profiles in the green tissues were similar between treated and non-treated plants. However, in the roots, the total content of lipids increased significantly including sterols (L11/R6 spot in Figure 2(A)), and several new spots (-Rs3) appearedn the H_2_O_2_-treated roots, suggesting H_2_O_2_ has strong impacts on root lipid profiling. To test the dosage effect of H_2_O_2_ on leaf and root lipid profiles, we treated the plants with H_2_O_2_ at the different concentrations for 2 (Figure 2(B)) and 4 days (Figure 2(C)), respectively, and found that the increased lipid spots were also correlated with the H_2_O_2_ concentration (Figure 2(C)). Nevertheless, some spots, e.g. Rs2, 4, 5, 6 spots were only detected in 7-day treated plants (Figure 2(A)), rather than in 2- (Figure 2(B)) or 4-day treated plants (Figure 2(C)).

To further confirm that the increase in root lipids was caused by H_2_O_2_ treatment, we used a plasma membrane-marked mCherry (AtPIP2a-mCherry) transgenic line to monitor the lipid alterations. Remarkably, the fluorescence signal was strongly increased after 24 h treatment (Figure 2(E)) compared with the control (Figure 2(D)), exactly recapitulating the TLC results. To assess how the membrane fluorescence was increased after H_2_O_2_ treatment, we used a fungal toxin BFA to inhibit vesicle or lipid exocytosis [8] to check if this could alter fluorescence signal. Remarkably, H_2_O_2_-induced membrane fluorescence increase was blocked by BFA (Figure 2(F)). The fluorescence pattern was changed into dots-like aggregates known as BFA-induced compartments or BFA-induced endocytosis effect (inset in Figure 2(F)). Nevertheless, this effect seemed to be reversed (Figure 2(G)) when the pretreated plants with H_2_O_2_ and BFA were further treated with auxin naphthalene-1-acetic acid (NAA), agreeing with previous work [9,10]. Together, these results suggested that the increased membrane fluorescence due to exocytosis could be correlated with the increased lipids induced by H_2_O_2_.

### Dramatic changes of lipid profiles to various ROS inhibitors

**Figure 3.**
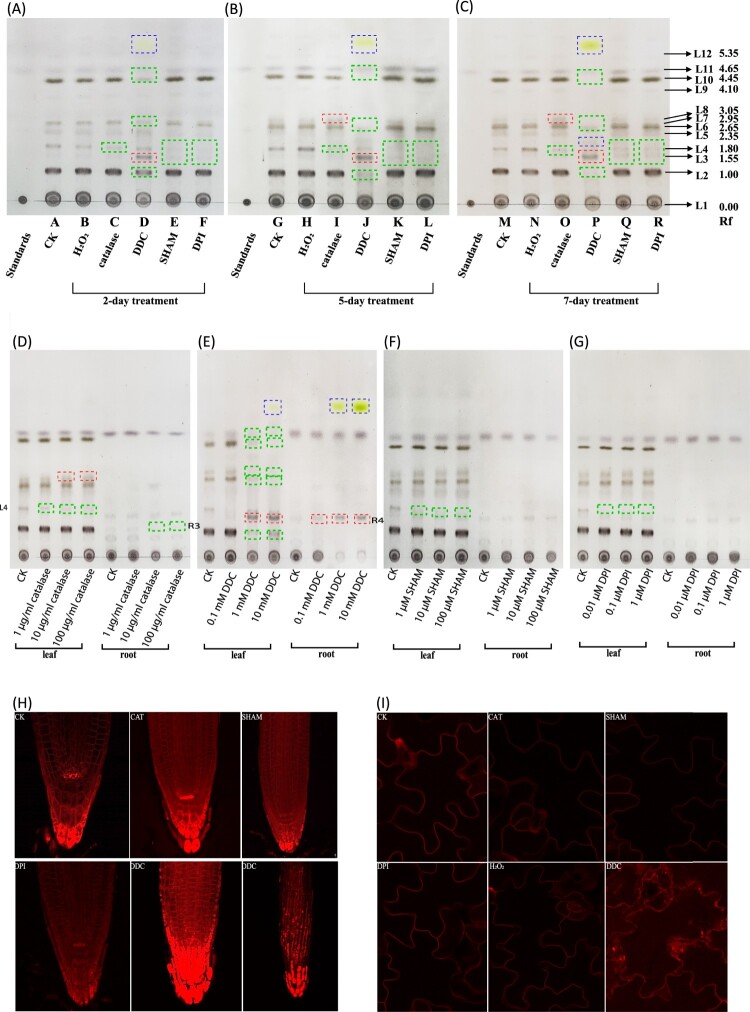
Lipid profile and plasma membrane marker response to ROS inhibitors. Lipid profiles of whole plant materials after 2-day treatment (A), 5-day treatment (B) and 7-day treatment (C). A noticeable change occurred at 5-day treatment by catalase (lane I & lane O). L4 spot decreased and L8 spot increased significantly compared to control. The most significant changes occurred by DDC treatment (lane D, J and P). L3 spot increased significantly. L2, L4, L5, L6, L7, L8, L9, L10, L11 spots gradually decreased with longer treatment. Three new spots were found (blue dashed box) in DDC-treated samples. L3 spot increased and L4 spot decreased significantly by SHAM and DPI compared to CK. (D-G) Dose-dependent effect of catalase (D), DDC (E), SHAM (F) and DPI (G) on the leaf and root lipid profiles after 2-day treatment. Green dotted box indicates decreased spots. Red dotted box indicates increased spots. Blue dotted box indicates new spots after treatment. (H) Plasma membrane marker was responding to catalase, SHAM, DPI and DDC. Catalase increased membrane marker signal mainly in the RAM whereas SHAM decreased membrane marker signal in the root apical meristem. DPI decreased membrane marker signal compared to control. DDC can significantly increase membrane marker signal in the root tips (bottom middle) and epidermal cells (bottom right). (I) Plasma membrane marker in the leaf was responding to catalase, SHAM, DPI and DDC. Fluorescence signal was increased in the leaf treated by DDC. No significant changes were observed by catalase, SHAM or DPI. Each figure was representative of three experiments with replicates.

We next asked whether perturbations of ROS balance through chemical administration could induce any changes in lipid profiles in the leaves. To do this, we used catalase for external H_2_O_2_ depletion, diphenyleneiodonium (DPI) for inhibiting NAD(P)H oxidase, 2′-3′-dideoxycytidine (DDC) for inhibiting superoxide dismutase and salicylhydroxamic acid (SHAM) for inhibiting peroxidase. After 2-day treatment, the plants treated with DPI and SHAM displayed a reduced lipid accumulation at L3, L4 spots (Figure 3(A)). DDC-treated plants showed stronger increase in L3 spot and generated new spots including one that was stained in yellow (Figure 3(B,C,E)), and most obviously, DDC caused significant reduction in the major spots L2, L7, L8, L10 and L11 (Figure 3(B,C)). To further confirm this is not an artifact, we performed TLC experiments on plants treated after 5 days (Figure 3(B)) and 7 days (Figure 3(C)). The trend of lipid changes appeared to be more obvious in these longer treatment situations. The L2, L7 and L10 spots were gradually decreased (Figure 3(B)) and reduced beyond detection after 7-day treatment (Figure 3(C)) in the DDC-treated plants, exactly opposite to the L3 spot. For plants treated with catalase, L4 spot decreased and L8 spot slightly increased compared to control (Figure 3(B,C)).

To explore whether these altered spots were specifically responding to these ROS-altering reagents, we subjected *Arabidopsis* plants to different doses of these reagents. After 2-day treatment, the L4 spot from leaf and R3 from roots were gradually decreased with the increase of catalase concentration (Figure 3(D)). Similarly, L4 spot was all reduced in DDC, DPI and SHAM treatments (Figure 3(E,F,G)). L2, L7, L8 and L10 spots were all reduced in the leaves when plants were subjected to higher concentration of DDC (e.g. 1 and 10 mM) (Figure 3(E)). L3 spot and R4 were gradually increased in both leaves and roots (Figure 3(E)). The newly appearing yellow spot was detected apparently in a dose-dependent way in roots, but only slightly detected in the shoots under higher DDC concentration, i.e. 10 mM (Figure 3(E)), suggesting this spot was mainly induced in roots by DDC. These results indicated the changes of some spots were specifically responding to ROS perturbations.

We then checked the membrane marker under these drugs’ treatment. The fluorescence was slightly reduced by DPI and SHAM (Figure 3(H)). Interestingly, the fluorescence in DDC-treated plants was strongly induced in both leaves and roots (Figure 3(H,I)). Different from the H_2_O_2_ treatment, the increased fluorescence was mainly confined in the root cap and the epidermal tissues (Figure 3(H)), suggesting a different role of DDC in interfering membrane lipids. Catalase also has a positive effect on the membrane lipids but only around the root apical meristem (RAM) (Figure 3(H)). Except DDC, these drugs have no obvious impact on the fluorescence pattern in the leaf epidermal cells (Figure 3(I)).

## Discussion

In plants and animals, lipid distribution in tissues appears very diverse such that different tissue expressed a unique lipid profile [11–13]. By using a TLC method, we found that roots differed greatly from that of leaves (Figure 1 and Figure 2(A,B)), agreeing with the recent findings in *Arabidopsis* [11]. Actually, in many plant species, the lipid composition differs significantly between roots and leaves [14–16]. The higher lipid abundance in leaves may be related to the fact that the photosynthetic organs require different lipids for the stability of photosynthetic complexes [5,17,18]. Interestingly, the sterol content in the root is slightly higher than in the leaves, agreeing with the findings that sterols are critical for both root initiation and growth [19].

The distribution of lipids in different tissues could imply that different tissues responded to external signals with a different outcome given the lipids in the membranes are at the front line of receiving external signals. This is particularly true for H_2_O_2_. The roots, rather than the leaves are responding strongly to external H_2_O_2_ supplement (Figure 2(A,C)) such that some lipids were significantly increased. Interestingly, in marine microalgae *Phaeodactylum tricornutum*, a recent study also showed that H_2_O_2_ treatment greatly increased the total levels of neutral lipids [20]. The mechanism by which H_2_O_2_ enhances lipid production in these experiments remains largely unknown. From the perspective of oxidative stress, the increased fatty acid biosynthesis may provide protective function by acting as an electron sink to mitigate ROS damage during lipid synthesis [21,22].

In addition, H_2_O_2_ could act as beneficial factor that was used to establish intercellular channel [3]. We noticed that the increased membrane signals were mainly confined in the conducting part of roots, i.e. the cortex and pith region (Figure 2(E)), implying that the differently deposited lipids in the root cells could be associated with physiological transport and/or communication. We previously showed that external H_2_O_2_ supplement could accelerate intercellular movement of small RNA-mediated gene silencing [23]. Since the region where the increased lipids were deposited was the central part of roots mainly responsible for long-distance transport, we concluded that the H_2_O_2_-induced lipid deposition was likely to facilitate intercellular flow as previously proposed [3].

Meanwhile, it would be noteworthy, in this experiment, that the plasma membrane was marked by AtPIP2a-mCherry fusion that could be induced by H_2_O_2_ to relocate from the plasma membrane to intracellular compartments [10], resulting in the reduced efficiency for monitoring lipids. Nevertheless, H_2_O_2_-induced AtPIP2a-mCherry internalization does not overturn the enrichment of membrane lipids in the root conducting region (Figure 2(E)), further supporting the role of H_2_O_2_ in membrane enrichment and formation, thus in intercellular communication [3]. Another limitation is that the dose of hydrogen peroxide applied in this study might not necessarily reflect the impact of internal peroxide production on lipid profiling in most cases. But under certain circumstances, this situation was still possible; for example, H_2_O_2_ could rapidly accumulate to a very high level (millimolar concentration) in soybean cells during oxidative burst [24], presumably leading to lipid alterations. Whether the lipid profiles were responding to internal peroxide change under normal or mild stress conditions awaits further investigation.

Results from H_2_O_2_ treatment further raise questions of whether other redox perturbation regents could lead to the changes in lipid profiles. Using a similar strategy, we found different ROS inhibitors could give rise to different results; both SHAM and DPI reduced lipid reduction at L3, L4 spots. DDC strongly increased L3 spot but reduced L2, L7 and L10 spots. DPI slightly decreased the membrane marker signal, agreeing with the reduced lipid spots. Interestingly, DDC can strongly increase the membrane accumulation in both leaves and roots (Figure 3(H,I)). Different from the H_2_O_2_-induced effects, DDC-induced membrane marker increase was mainly occurring in the root tips and the epidermal cells, suggesting that specific tissue-derived lipids were reacting differently to ROS. This could be due to the differential lipid constituents of root tissues; that is, the epidermal cells have lower lipid order than the cortical cells in the roots [25]. The lower lipid order in the epidermal cells might be more sensitive to ROS-induced (or in this case DDC-induced superoxide) lipid peroxidation as shown in animal cells [26], thus responding actively with a higher lipid accumulation in the membranes of these tissues. Further experimental designs are needed to tease out how H_2_O_2_ or superoxide can cause extensive changes of lipid profiles.

## Conclusion

Lipid profiles in different parts of *Arabidopsis* plants were responding differently to ROS. We found lipids in the roots, rather than in the leaves, were more responsive to H_2_O_2_ treatment. Superoxide-inducing agent DDC could significantly cause profile changes in the leaves. Our results provided clear clues for investigating ROS-induced membrane dynamics in plant tissues.

## Data Availability

The data have analyzed during the current study are available from the corresponding author on reasonable request.
